# The complete mitochondrial genome of *Leuciscus merzbacheri* (Cypriniformes: Cyprinidae)

**DOI:** 10.1080/23802359.2023.2189496

**Published:** 2023-03-20

**Authors:** Wei Meng, Lin Li, Xiaoqian Yuan, Yongdong Zhou

**Affiliations:** a Zhejiang Marine Fisheries Research Institute, Key Laboratory of Sustainable Utilization of Technology Research for Fisheries Resources of Zhejiang Province, Scientific Observing and Experimental Station of Fishery Resources for Key Fishing Grounds, Ministry of Agriculture, Zhoushan, China; b Xinjiang Fisheries Research Institute, Urumqi, China; c College of life Science and Technology, Xinjiang University, Urumqi, China

**Keywords:** Leuciscinae, mitochondrial genome, *Leuciscus merzbacheri*, phylogeny

## Abstract

*Leuciscus merzbacheri* (Zugmayer, 1912) is a cyprinid fish endemic to China, with a distribution range limited to Xinjiang Province. As a landmark species in the Junggar Basin, *L. merzbacheri* is of considerable significance regarding our understanding of the adaptive evolution of salt and alkali tolerance. In this study, the complete mitochondrial sequence of *L. merzbacheri* was obtained for the first time by high-throughput sequencing. The circular mitogenome is 16,609 bp in length and contains the standard 37 genes, including 13 protein-coding, 22 transfer RNA, and 2 ribosomal RNA genes, which is similar to that of other fish. The mitogenome contents of A, T, C, and G were 27.9, 26.3, 27.1, and 18.7%, respectively. Phylogenetically, *L. merzbacheri* was located on a new branch near the base of the phylogenetic tree, thereby suggesting an early origin.

## 1. Introduction

In terms of dominant species and quantity, cyprinid fishes are among the most important freshwater fish inhabiting the inland waters of China. Within the family Cyprinidae, fish in the subfamily Leuciscinae include a large number of economic significance. To date, more than 20 species of Leuciscinae have been described, a majority of which are distributed in Europe and western and northern Asia (Wu and Wu 1992). At present, six Leuciscinae species in China can be found in the Yellow River and its northern water system, among which, *Leuciscus merzbacheri* is a species endemic to Xinjiang, wherein is has been reported in a number of rivers systems, including the Bortala, Manas, Urumqi rivers (Guo et al. 2012), and has been designated as a landmark species in the Junggar Basin. Although once the main economic fish in Xinjiang, since the 1950s, the large-scale development of northern Xinjiang, overfishing, and destruction of water resources have contributed to a marked decline in the natural resources of *L. merzbacheri*. In 1998, this fish was listed as a vulnerable species in the Red Book of endangered animals in China, and in 2004, it was listed among the Grade II protected aquatic wild animals by the Xinjiang government. However, recent breakthroughs in the field of artificial reproduction have been conducive to the protection and restoration of *L. merzbacheri* resources.

## 2. Materials and methods

### 2.1. Sample collection and preservation

**Figure 1.**
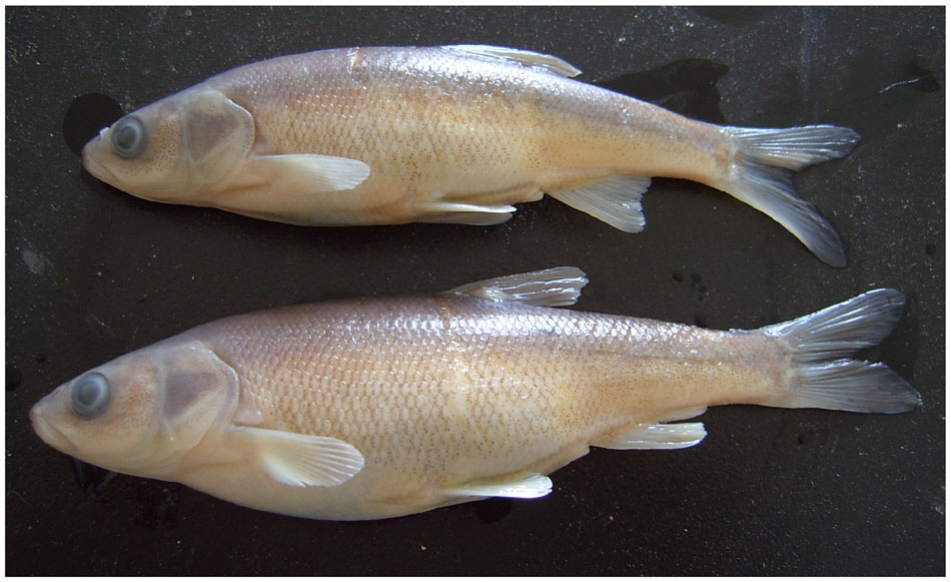
Picture of *L. merzbacheri* specimen (cited from ‘Fish Fauna of Xinjiang,’ Yan Guo et al. 2012).

A specimen of *L. merzbacheri* (Figure 1) was collected from Urumqi (87°34′E, 44°15′N). Identification was based on the criterion of a number of lateral scales greater than 65. The voucher specimen (Sample No.: FS-20190918; zfcxju@xju.edu.cn) has been deposited in the Key Laboratory of Biological Resources and Genetic Engineering, Xinjiang University. For the purposes of sampling, fins were detached, immersed in 95% alcohol, and stored at −20 °C for subsequent analysis.

### 2.2. DNA extraction, sequencing, and assembly

Genomic DNA was extracted from fin tissues using the phenol–chloroform method (Sambrook et al. 1982). A DNA library was sequenced using the Illumina HiSeq2500 platform, which yielded a total of 6.39 Gb of raw data. Having performed sequencing quality control, the raw data were filtered, and the adaptor sequence and low-quality reads were removed, thereby yielding 6.37 Gb of high-quality clean data. The percentage of Q30 bases in the sample was not less than 90%. The mitochondrial genome was directly assembled using MitoZ software (Meng et al. 2018) with default parameters.

### 2.3. Annotation and analysis

**Figure 2.**
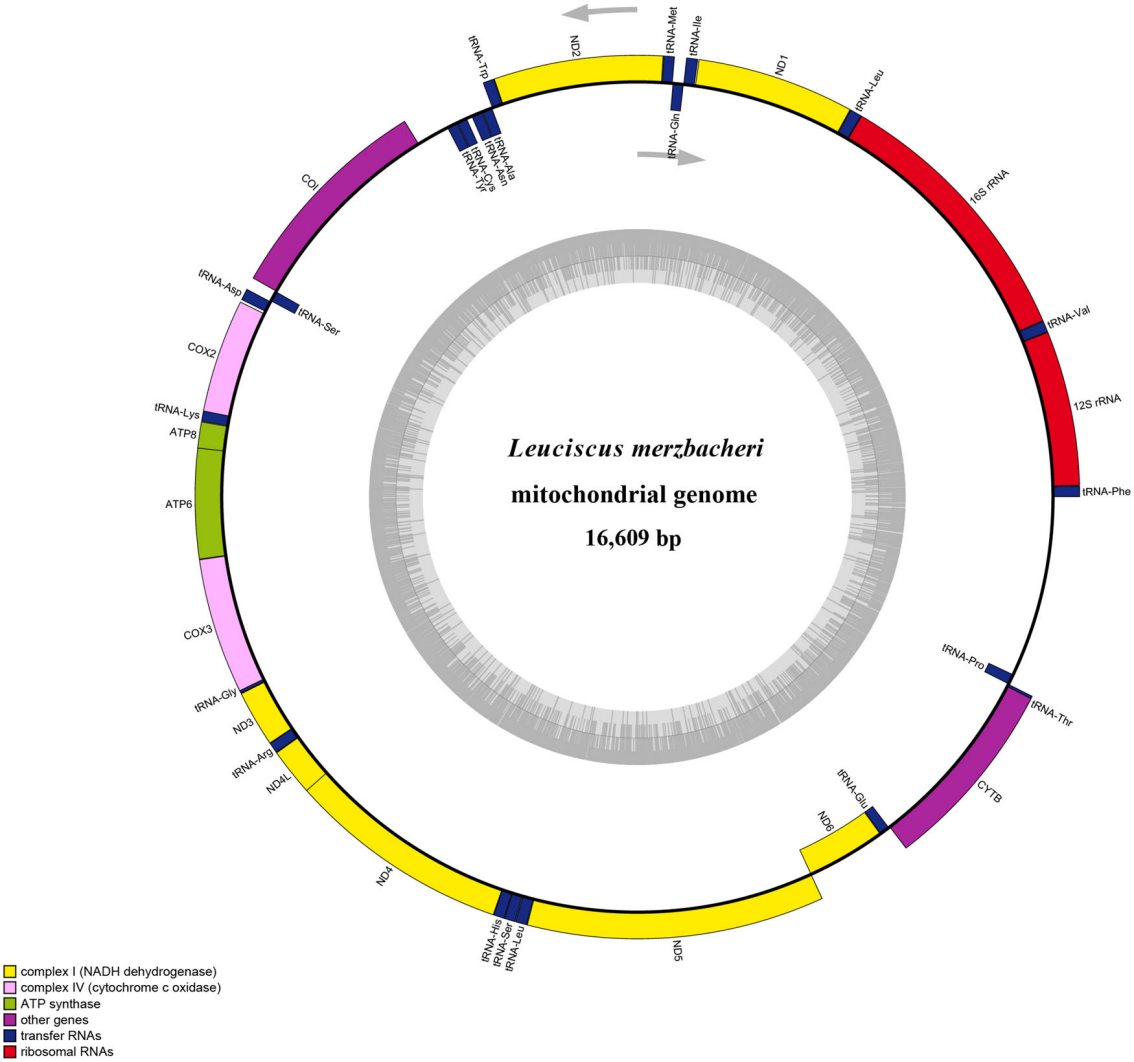
Genome map of *L. merzbacheri.*

As a reference mitochondrial sequence, we used that of *Leuciscus baicalensis* (NC_024528.1). The mitochondrial genome was annotated online using MitoFish software (Xuan et al. 2013) (Figure 2). Phylogenetic analysis was performed based on multiple alignments of protein-coding sequences in the mitochondrial genomes of 11 Cyprinidae fish species using MEGA7 (Kumar et al. 2016). A phylogenetic tree was constructed based on the neighbor-joining (NJ) method using the Kimura 2-parameter model.

## 3. Results and discussion

### 3.1. Genomic characterization

The complete mitochondrial genome of *L. merzbacheri* is 16,609 bp (ON457584) in length and comprises 37 genes, namely 13 protein-coding (PCG), 22 transfer RNA (tRNA), and 2 ribosomal RNA (rRNA) genes. The contents of A, T, C, and G in the mitochondrial genome were found to be 27.9, 26.3, 27.1, and 18.7%, respectively, with the proportion of A + T being greater than that of C + G, thus indicating a clear anti-G bias. In terms of the PCG initiation codons, there are 11 ATG (*ND1, ND2, ND3, ND4, ND4L, ND5, COX2, COX3, ATP6, ATP8*, and *CYTB*), one GTG (*COX1*), and one TTA (*ND6*). Among the termination codons, there are five TAG (*ND1, ND2, ND3, ND5, ATP8*), four TAA (*COX1, ATP6, ND4L, ND6*), three T (*COX2, COX3, CYTB*), and one TA (*ND4*).

### 3.2. Phylogenetic analysis

**Figure 3.**
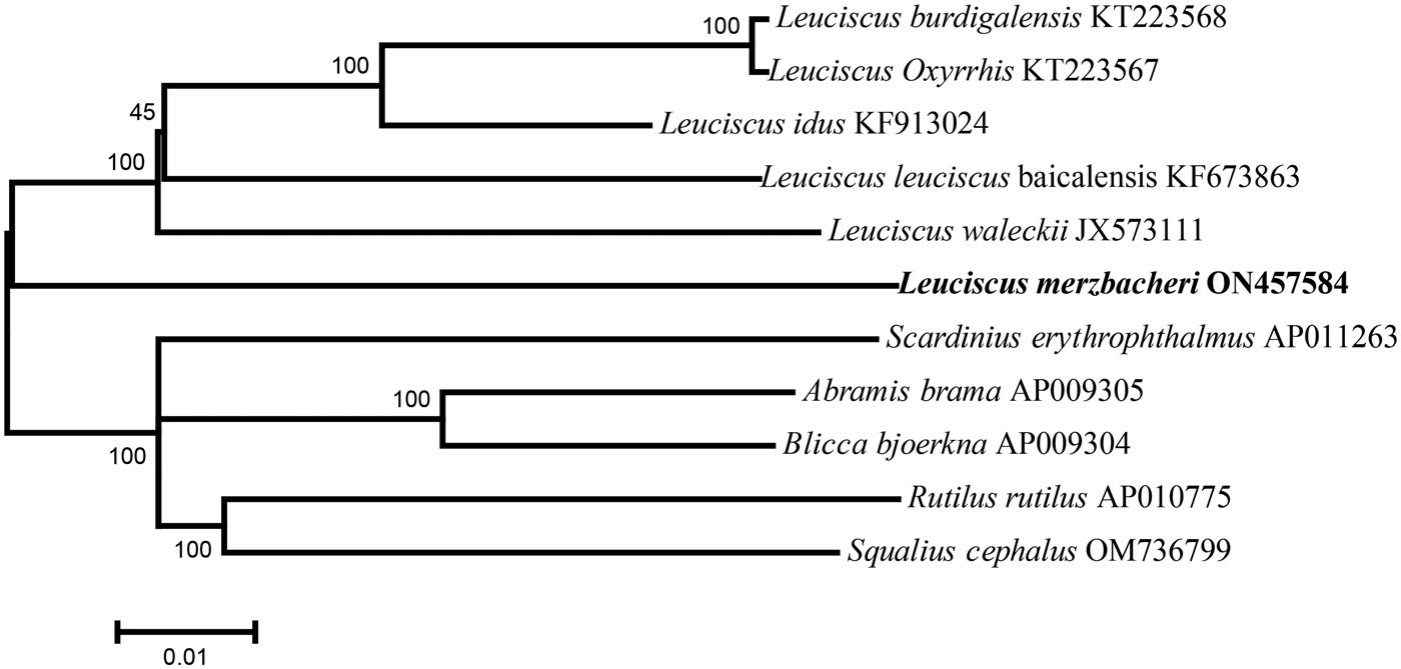
Phylogenetic tree obtained from 7 mitogenome sequences based on 13 PCGs. The following sequences were used: KT223568 (Hinsinger et al. 2015), KT223567 (Hinsinger et al. 2015), KF913024, KF673863, JX573111(Wang et al. 2013), AP011263, AP009305 (Imoto et al. 2013), AP009304 (Imoto et al. 2013), AP010775, OM736799 .

To clarify the phylogenetic position of *L. merzbacheri,* we performed analysis using 11 species of Cyprinidae fish, with five species being used as an outgroup (Figure 3). In the phylogenetic tree, *L. burdigalensis* and *L. oxyrrhis*, which are both distributed in Europe, were found to cluster one branch, thereby indicating a close relationship, and these are slightly more distantly related to *L. idus*, which is distributed in Europe and Asia. *L. baicalensis* and *L. waleckii*, which are distributed in Asia, cluster separately from these species, and *L. merzbacheri*, a species endemic to Xinjiang, China, was found to be located at an outer-most base. This clustering pattern provides evidence to indicate that *L. merzbacheri* is a relatively primitive species in the genus *Leuciscus*, and that other species in this genus may have spread east and west from the Xinjiang source, and subsequently evolved independently into the current-day species. Among the other *Leuciscus* species, *L. waleckii*, which is distributed in Northeast China, has received considerable attention with respect to its physiological characteristics of salt and alkali tolerance (Xu et al. 2017). Accordingly, we speculate that by establishing the evolutionary relationships between *L. merzbacheri* and *L. waleckii*, we may be able to gain a better understanding of the mechanisms underlying the evolutionary adaptation of *Leuciscus* fish to water salinity and alkalinity, as well as further insights into the biogeography of *L. merzbacheri.*

## 4. Conclusion

The complete mitochondrial genome of *L. merzbacheri* was sequenced using the Illumina Hiseq platform, generating a 16,609-bp mitogenome (Genbank accession no. ON457584). Phylogenetic analysis provided evidence to indicate that *L. merzbacheri* may represent an early species in the evolutionary origin of the genus *Leuciscus*. These findings will provide a basis for further research on the phylogeny, adaptive evolution, and genetic resources conservation of *Leuciscus* fish.

## Data Availability

The genome sequence data that support the findings of this study are openly available in GenBank of NCBI at https://www.ncbi.nlm.nih.gov/ under the accession no. ON457584. The associated BioProject, SRA, and Bio-Sample numbers are PRJNA855547, SRR19974985 and SAMN29494530, respectively.
